# The role of chemotherapy in the treatment of stage II nasopharyngeal carcinoma: Retrospective analysis of the national cancer database

**DOI:** 10.1002/cam4.2033

**Published:** 2019-02-21

**Authors:** Zaheer Ahmed, Lara Kujtan, Kevin Kennedy, Valerie Wood, David Schomas, Janakiraman Subramanian

**Affiliations:** ^1^ Department of Medicine University of Missouri Kansas City Missouri; ^2^ Department of Cardiovascular Research Saint Luke's Hospital Kansas City Missouri; ^3^ Department of Otolaryngology Saint Luke's Hospital Kansas City Missouri; ^4^ Department of Radiation Oncology Saint Luke's Hospital Kansas City Missouri; ^5^ Division of Oncology Saint Luke's Cancer Institute Kansas City Missouri; ^6^ Center for Precision Oncology Saint Luke's Cancer Institute Kansas City Missouri

**Keywords:** concurrent chemoradiotherapy, nasopharyngeal carcinoma, overall survival, radiotherapy, stage II

## Abstract

The standard of care treatment for locally advanced nasopharyngeal carcinoma (NPC) includes both chemotherapy and definitive radiation. However, there are limited data on the optimal management of stage II NPC. We performed a retrospective analysis of the National Cancer Database to analyze the treatment patterns and role of chemotherapy in patients with stage II NPC. We identified 611 patients diagnosed with T1‐2, N0‐1, M0 NPC, from 2004 to 2013. Five‐year survival was calculated using Kaplan Meier (KM) analysis. Multivariable analysis and propensity matched analysis were performed to analyze the impact of chemotherapy on overall survival. Of the 611 patients, 527 underwent concurrent chemoradiation (CCRT) and 84 received radiation only. Unadjusted KM analysis showed improved 5‐year survival in the CCRT group compared to radiation only (80.5% vs 65.7%; *P* = 0.0021). Multivariable analysis also showed improved survival with the addition of chemotherapy (Hazard ratio [HR] 0.59; 95 CI 0.39‐0.89; *P* = 0.0124). Propensity matched analysis confirmed a significant clinical benefit from the addition of chemotherapy to radiation. Age ≥ 65 years (HR 2.41; 95% CI 1.71‐3.4; *P* = <0.0001), Charlson‐Deyo comorbidity index >1 (HR 2.82; 95% CI 1.49‐5.31; *P* = 0.0014) and positive lymph node status (HR 1.6; 95% CI 1.04‐2.46; *P* = 0.0340) were associated with worse survival. In this retrospective analysis, patients with stage II NPC had improved survival with CCRT compared to definitive radiation only. Elderly patients with comorbidities had worse outcomes.

## 1 INTRODUCTION

Nasopharyngeal carcinoma (NPC) is a highly radiosensitive tumor and radiotherapy (RT) is the mainstay of treatment for early stage disease.^1^ The role of chemotherapy is well established for the treatment of locally advanced nasopharyngeal carcinoma and multiple studies have shown improved overall survival with combined chemoradiation.^2^, ^3^, ^4^, ^5^, ^6^, ^7^, ^8^ The National Comprehensive Cancer Network (NCCN) guidelines also recommend the use of concurrent chemoradiation (CCRT) and adjuvant chemotherapy for the treatment of locally advanced nasopharyngeal carcinoma.^9^ However, the role of chemotherapy for the treatment of stage II has not been well studied. A single phase III trial in Chinese patients with stage II NPC showed improved overall survival with CCRT compared to RT alone.^10^ To our knowledge there are no similar types of data available to guide treatment of patients with stage II NPC in the United States. Even more importantly, we do not know the impact of demographics and clinical features on treatment outcomes in stage II NPC. Having access to that information can guide selection of the appropriate treatment in an individual patient. Therefore, we undertook this study to investigate the treatment patterns and survival outcomes in North American patients with stage II NPC and to evaluate the role of chemotherapy in the treatment of this population.

## 2 MATERIALS AND METHODS

The National Cancer Database (NCDB) is a joint project of the Commission on Cancer (CoC) of the American College of Surgeons and the American Cancer Society. The NCDB is a nationwide and facility‐based oncology dataset that currently captures 70% of all newly diagnosed malignancies in the US annually.^11^ Currently the NCDB collects data from over 1500 participating hospitals.^12^ The NCDB Participant Use Data File (PUF) for head and neck cancers was used to identify all the cases in the study. The NCDB PUF is a Health Insurance Portability and Accountability Act (HIPAA) compliant data file which contains de‐identified patient level data that do not identify hospitals, healthcare providers, or patients. It is a publicly available dataset without any protected health information (PHI) and not considered to be human subjects research. The study was reviewed by the ethics committee at our institution and it was classified as exempt. The American College of Surgeons and the CoC have not verified and are not responsible for the statistical validity of the data analysis or the conclusions derived by the authors.

**Figure 1:**
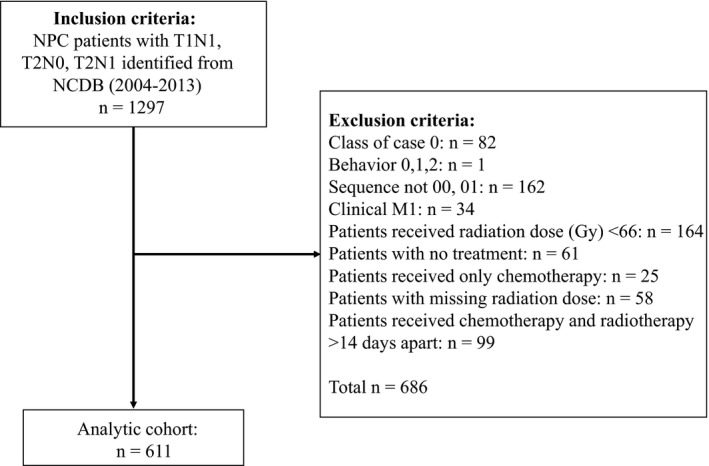
Inclusion and exclusion criteria for the study population

The American Joint Committee on Cancer (AJCC) 6th and 7th editions were used for staging. From 2004 to 2013, we identified patients diagnosed with nasopharyngeal carcinoma with clinical stage T1N1, T2N0, T2N1 from the NCDB by using International Classification for Disease for Oncology (ICD‐O‐3) topographic and histology codes. Patients were identified by using following histology codes for NPC: 8052, 8070, 8071, 8072, 8073, 8074, 8083 which corresponded to papillary squamous cell carcinoma, squamous cell carcinoma not otherwise specified (NOS), keratinizing squamous cell carcinoma NOS, nonkeratinizing squamous cell carcinoma (large cell), nonkeratinizing squamous cell carcinoma (small cell), squamous cell carcinoma (spindle cell), basaloid squamous cell carcinoma, respectively. We excluded nonsquamous and unknown tumor histology. The following topographic codes were used: C11.0, C11.1, C11.2, C11.3, C11.8, and C11.9 which corresponded to superior wall of nasopharynx, posterior wall of nasopharynx, lateral wall of nasopharynx, anterior wall of nasopharynx, overlapping lesion of nasopharynx, nasopharynx (NOS), respectively. Concurrent chemoradiation was defined as chemotherapy start date occurring ≤14 days before or after the start date of radiation similar to a previously published study evaluating radiation treatments in patients with head and neck squamous cell carcinoma.^13^ Patients receiving chemotherapy outside this 14 day window were excluded from the analysis. Current NCCN guidelines recommend definitive radiation dose of 66 Gy–70 Gy for patients with NPC.^9^ We excluded patients who received <66 Gy total radiation treatment. We also excluded patients who received only chemotherapy, patients with distant metastasis, patients who did not receive any treatments and with missing radiation dose (Figure 1). The final analytic cohort (n = 611) was divided into two treatment groups; CCRT and RT alone.

Patient demographics included age at the time of diagnosis, gender, race, insurance status, income, education status, Charlson‐Deyo comorbidity index (CDCI), and radiation dose (Gy) are shown in Table 1. These were compared with Student's *t*‐test and chi‐square tests for univariate associations. Significance was defined as a *P* value < 0.05. To isolate the effect of chemotherapy on mortality, we performed a multivariable proportional hazards model adjusted for: age, gender, ethnicity, tumor grade, zip‐code income, zip‐code education, Charlson‐Deyo comorbidity index, treatment center, nodal status (clinical), clinical T stage, IMRT, and radiation dose. The results were reported as hazard ratios (HR) with 95% confidence intervals (CI). Five‐year survival between the CCRT and RT alone groups were calculated using Kaplan Meier (KM) analysis. Since selection bias is a significant limiting factor in retrospective cohort analysis, we performed a propensity matched pair analysis to compare 5‐year survival between CCRT and RT. To control for immortal time bias, we performed a 6‐month landmark analysis for all survival estimates. All analyses were performed with SAS 9.4 (Cary, NC).

**Table 1:** Patient demographics

Variable	Radiation alone N (%) 84 (13.8)	Concurrent chemoradiation N (%) 527 (86.2)	Total N (%) 611 (100)	*P* value
Median age at diagnosis (years)	64.4 ± 13.3	56.0 ± 11.8	57.2 ± 12.3	<0.0001
Gender				
Male	60 (71.4)	359 (68.1)	419 (68.6)	0.5442
Female	24 (28.6)	168 (31.9)	192 (31.4)	
Ethnicity				
White	59 (70.2)	338 (64.1)	397 (65)	0.5523
African‐American	10 (11.9)	60 (11.4)	70 (11.5)	
Asian	11 (13.1)	84 (15.9)	95 (15.5)	
Others	4 (4.8)	45 (8.5)	49 (8)	
Insurance status				
Private Insurance	34 (40.5)	320 (60.7)	354 (57.9)	0.0023
Medicare	35 (41.7)	118 (22.4)	153 (25)	
Medicaid	5 (6)	48 (9.1)	53 (8.7)	
Not Insured	5 (6)	20 (3.8)	25 (4.1)	
Other Government	3 (3.6)	15 (2.8)	18 (2.9)	
Insurance Status Unknown	2 (2.4)	6 (1.1)	8 (1.3)	
Treatment Center				
Community Cancer Program	7 (8.3)	63 (12)	70 (11.5)	0.7309
Comprehensive Community Cancer Program	40 (47.6)	232 (44)	272 (44.5)	
Academic/Research Program	28 (33.3)	184 (34.9)	212 (34.7)	
Integrated Network Cancer Program	9 (10.7)	48 (9.1)	57 (9.3)	
Median Income Quartiles 2008‐2012				
<$38 000	15 (17.9)	88 (16.7)	103 (16.9)	0.3241
$38 000‐$47 999	25 (29.8)	123 (23.3)	148 (24.2)	
$48 000‐$62 999	23 (27.4)	133 (25.2)	156 (25.5)	
≥$68 000	21 (25)	183 (34.7)	133 (25.2)	
No high school degree 2008‐2012 (%age)				
≥21	15 (17.9)	96 (18.2)	111 (18.2)	0.9608
13‐20.9	24 (28.6)	136 (25.8)	160 (26.2)	
7‐12.9	30 (35.7)	198 (37.6)	228 (37.3)	
<7	15 (17.9)	97 (18.4)	112 (18.3)	
Charlson‐Deyo comorbidity index (CDCI)				
0	69 (82.1)	438 (83.1)	507 (83)	0.9691
1	12 (14.3)	70 (13.3)	82 (13.4)	
2	3 (3.6)	19 (3.6)	22 (3.6)	
Clinical stage				
T1‐N1	27 (32.1)	214 (40.6)	241 (39.4)	<0.0001
T2‐N0	44 (52.4)	111 (21)	155 (25.3)	
T2‐N1	13 (15.5)	202 (38.4)	215 (35.2)	
Total radiation dose (Gy)	71.0 ± 4.3	70.7 ± 3.8	70.8 ± 3.9	0.5491
TNM Edition				
Sixth Edition	57 (67.9)	282 (53.5)	339 (55.5)	0.0140
Seventh Edition	27 (32.1)	245 (46.5)	272 (44.5)	
Vital status				
Alive	49 (58.3)	405 (76.9)	454 (74.3)	0.0003
Dead	35 (41.7)	122 (23.1)	157 (25.7)	

## 3 RESULTS

**Figure 2:**
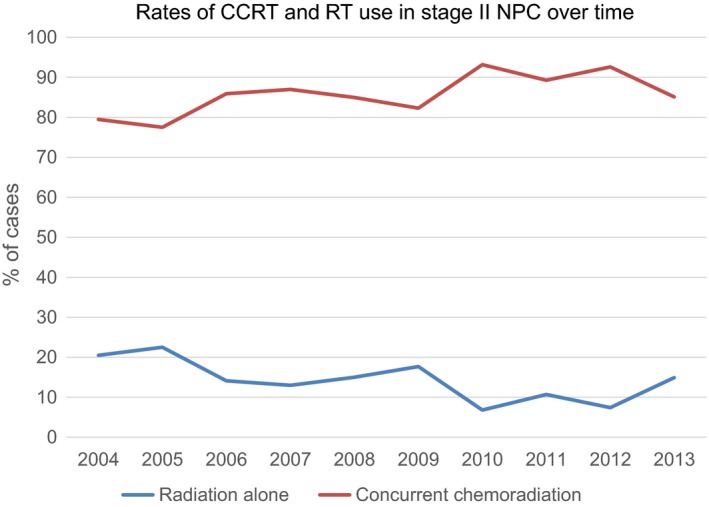
Rates for use of concurrent chemoradiation and radiation in stage II nasopharyngeal carcinoma patients in the United States over time (2004‐2013). The proportion of patients receiving concurrent chemoradiation did not change significantly over time (*P* = 0.2314)

We identified 611 patients with NPC who met our pre‐specified inclusion and exclusion criteria (Figure 1). Of these patients, 419 (68.6%) were male. The majority of patients were white 397 (65%), followed by Asians 95 (15.5%) and African‐Americans 70 (11.5%). Three hundred fifty‐four patients (57.9%) had private insurance followed by Medicare 153 (25%) and Medicaid 53 (8.7%). Median age at diagnosis was 57.2 years (range 44.9‐69.5 years). The two groups were similar in demographics and clinical characteristics, except patients who received CCRT were younger compared to the RT alone group (median age 56 years vs 64.4 years; *P* < 0.0001). In addition, patients with private insurance (60.7%; *P* = 0.0023) and lymph node positive disease [n = 416 (78.9%) vs n = 40 (47.6%); *P* = <0.0001] were more likely to receive CCRT (Table 1). The proportion of patients receiving CCRT in this cohort did not change significantly over time (Figure 2; *P* = 0.2314).

**Figure 3:**
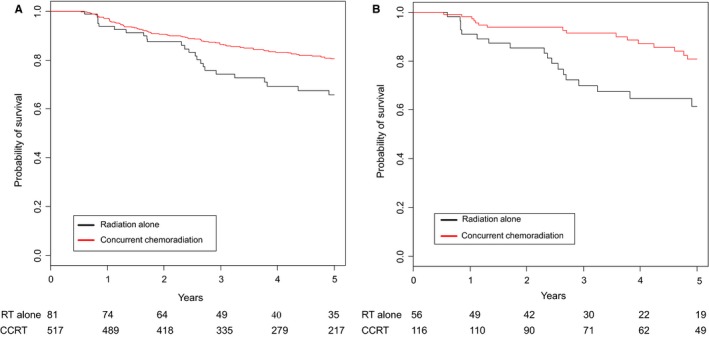
(A) Unadjusted Kaplan Meier curve for overall survival in concurrent chemoradiation and radiation alone groups showed statistically significant improved survival in concurrent chemoradiation group compared to radiation alone (80.5% vs 65.7%, *P* = 0.0021). (B) Propensity matched pair analysis for overall survival in concurrent chemoradiation and radiation alone groups showed statistically significant improved survival in concurrent chemoradiation group compared to radiation alone (80.7% vs 61.4%, *P *= 0.0074)

Eighty‐four patients (13.8%) received radiotherapy alone and 527 (86.2%) received CCRT. The median follow up time was 4.2 years. Four hundred fifty‐four (74.3%) patients were alive at the time of analysis, 58.3% in radiation alone group and 76.9% in CCRT group. Unadjusted KM analysis showed improved 5‐year survival in the CCRT group as compared to RT only (80.5% vs 65.7%; *P* = 0.0021; Figure 3A). Concurrent chemoradiotherapy improved overall survival across all stage groups (T1N1, T2N0, T2N1) though in patients with T2N0 stage it did not reach statistical significance (Table 2). Multivariable analysis (Table 3) showed improved survival with the addition of chemotherapy (HR 0.59; 95% CI 0.39‐0.89; *P* = 0.0124). Asian patients showed a trend toward improved overall survival compared to whites, but it did not reach statistical significance (HR 0.65; 95% CI 0.36‐1.18; *P* = 0.1568). Grade 3 and 4 tumors had improved overall survival compared to grade 1 tumors (HR 0.59; 95% CI 0.4‐0.88; *P* = 0.0098 and HR 0.38; 95% CI 0.18‐0.78; *P* = 0.0083, respectively).

**Table 2:** Subgroup analysis of stage II NPC for 5 year survival in CRRT and RT alone groups

Clinical stage	5‐year overall survival in CCRT group (%)	5‐year overall survival in RT alone group (%)	*P* value
T1N1	79.2	48.9	0.0073
T2N0	80.6	76.0	0.1294
T2N1	77.6	53.9	0.0240

**Table 3:** Multivariable analysis for predictors of overall survival in stage II NPC patients

Variable	HR and 95% CI	*P* value
Age ≥ 65	2.41 (1.71‐3.4)	<0.0001
Chemotherapy	0.59 (0.39‐0.89)	0.0124
Male	1.39 (0.99‐1.96)	0.0573
Ethnicity		
African‐American vs White	1.05 (0.64‐1.74)	0.8341
Asian vs White	0.65 (0.36‐1.18)	0.1568
Other vs White	1.34 (0.74‐2.43)	0.3337
Tumor grade		
Grade 2 vs 1	0.82 (0.53‐1.29)	0.3947
Grade 3 vs 1	0.59 (0.4‐0.88)	0.0098
Grade 4 vs 1	0.38 (0.18‐0.78)	0.0083
Median income quartiles (2008‐2012)		
Q2 ($38 000‐$47 999) vs Q1 (<$38 000)	0.89 (0.55‐1.45)	0.6383
Q3 ($48 000‐$62 999) vs Q1 (<$38 000)	1.3 (0.8‐2.12)	0.2970
Q4 ($63 000+) vs Q1 (<$38 000)	1.05 (0.63‐1.74)	0.8512
Education quartiles: (%age no high school degree 2008‐2012)		
Q2 (13‐20) vs Q1 (≥21)	0.93 (0.6‐1.43)	0.7403
Q3 (7‐12.9) vs Q1 (≥21)	0.86 (0.49‐1.52)	0.6106
Q4 (<7) vs Q1 (≥21)	1.05 (0.63‐1.74)	0.8509
Charlson‐Deyo comorbidity index (CDCI)		
CDCI: 1 vs 0	1.23 (0.77‐1.97)	0.3888
CDCI: >1 vs 0	2.82 (1.49‐5.31)	0.0014
Treatment Center		
Comprehensive vs Community	1.51 (0.87‐2.62)	0.1440
Academic vs Community	1.16 (0.79‐1.71)	0.4388
Integrated vs Community	1.21 (0.69‐2.11)	0.5094
Nodal status (clinical)		
N1 vs N0	1.6 (1.04‐2.46)	0.0340
T stage (clinical)		
T2 vs T1	1.07 (0.73‐1.58)	0.7219
Radiation		
IMRT vs no IMRT	0.94 (0.67‐1.32)	0.7182
Radiation Dose (Gy)		
<70 vs ≥70	1.26 (0.86‐1.84)	0.2423

Multivariable analysis also identified that age ≥65 years (HR 2.41; 95% CI 1.71‐3.4; *P* < 0.0001), Charlson‐Deyo comorbidity index >1 (HR 2.82; 95% CI 1.49‐5.31; *P* = 0.0014) and patients with positive lymph node status (HR 1.6; 95% CI 1.04‐2.46; *P* = 0.0340) were associated with poor overall survival. CCRT still showed improved 5‐year survival in older patients with age ≥65 years (64.7% vs 49.1%; *P* = 0.0823), patients with Charlson‐Deyo comorbidity index >1 (76.9% vs 47.3%; *P* = 0.0168) and N1 status (80.3% vs 53.1%; *P* < 0.0001) compared to RT alone. Other factors including ethnicity, insurance status, education level, facility type, tumor grade 1, clinical T stage, use of IMRT, and radiation dose did not affect the overall survival significantly (Table 3).

To minimize the selection bias toward CCRT and to control for the differences between the patients’ individual characteristics, we propensity score matched patients receiving RT to patients receiving CCRT. In this analysis, CCRT was associated with significant improved overall survival compared to RT alone (80.7% vs 61.4%, *P* = 0.0074; Figure 3B).

## 4 DISCUSSION

Nasopharyngeal carcinoma is most common in Southeast Asia and China particularly Southern China but relatively uncommon in the United States.^14^, ^15^ In the United States, the incidence rates in white men and women are 0.5 and 0.2 per 100 000 person‐years, respectively.^14^ The standard of care treatment for early stage nasopharyngeal carcinoma is radiotherapy. The role of chemotherapy in the treatment of early stage nasopharyngeal carcinoma in the Unites States has not been studied. To the best of our knowledge, this is the first study analyzing the treatment patterns and outcomes in US population diagnosed with stage II NPC.

Our analysis shows that approximately 86% of all patients with stage II NPC in the United States are treated with CCRT. However, older patients and patients with node negative disease were more likely to receive radiotherapy alone. The addition of chemotherapy was associated with a significant improvement in 5‐year unadjusted survival when compared with radiation alone. Multivariable analysis also identified that addition of chemotherapy reduced the risk for mortality in this patient population (HR 0.59; 95% CI 0.39‐0.89; *P* = 0.0124; Table 3). The benefit from the addition of concurrent chemotherapy was further confirmed by propensity matched analysis.

This finding is consistent with majority of the Asian studies that showed benefit from the addition of chemotherapy.^10^, ^16^, ^17^, ^18^, ^19^, ^20^, ^21^ In a phase III randomized trial, Chinese NPC patients were randomly assigned to CCRT (n = 116) or RT alone (n = 114).^10^ The 5‐year overall survival (94.5% vs 85.8%, *P* = 0.007) and distant metastasis‐free survival (94.8% vs 83.9%, *P* = 0.007) were all significantly improved in the CCRT arm than in the RT arm. The number of chemotherapy cycles was the only independent factor that was associated with overall survival (OS), progression free survival (PFS) and distant control.^10^ A pooled analysis of two phase III trials evaluating induction chemotherapy followed by radiation showed significant improvement in overall survival (5‐year OS 79% vs 67%, *P* = 0.048) and distant metastasis‐free rates (86% and 74%, *P* = 0.0053) in early stage NPC (T1‐T2, N0‐N1) compared to radiation alone.^17^ Retrospective studies in this population have also shown benefit from the addition of chemotherapy to definitive radiation.^18^^,^ 19

Multivariable Cox proportional hazards model for predictors of overall survival identified that older patients (age ≥ 65 years), Charlson‐Deyo index >1 and lymph node positive disease to be associated with increased risk for mortality (Table 3). These findings are consistent with previously published data. A retrospective analysis of 138 stage II patients with NPC identified age >60 years was an important prognostic factor for overall survival.^20^ Chen et al. in a univariate and multivariate analysis showed that advanced age was associated with statistically significant decreased locoregional relapse‐free survival and failure‐free survival.^22^ Another retrospective study demonstrated that age ≥60 years and Karnofsky Performance Status (KPS) <70 were significantly associated with poor overall survival.^18^ Prior studies have shown N1 nodal status to be a predictor of poor survival as compared to no lymph node involved (N0).^23^, ^24^, ^25^ It is possible that older patients were less likely to receive CCRT due to higher incidence of comorbidities and poor performance status. However, data from our study show that these groups also benefit from the addition of chemotherapy.

Our analysis shows that addition of chemotherapy to definitive radiation is associated with significant improvement in overall survival. Apart from the retrospective nature of this study, our analysis has other limitations as well. Patients receiving radiation alone were older than the CCRT cohort and more likely to be node negative. It is possible that the radiation only treatment cohort may have been selected based on other unknown confounding variables such as performance status which is not collected by the NCDB. But multivariable analysis confirmed benefit from chemotherapy after controlling for these potential confounding factors. To further confirm the benefit of CCRT over definitive RT, we performed a propensity score matched analysis. Furthermore, a 6‐month landmark analysis on all survival estimates was done to limit the effect of confounding factors related to retrospective analysis. Despite the rigorous analysis, the benefit from CCRT was confirmed in our study. Another limitation is that NCDB does not provide information on the type of chemotherapy agent, dose, and the number of cycles of treatment received. But taken together, our analysis clearly shows that CCRT is superior to RT alone in stage II NPC.

In conclusion, our retrospective analysis of the NCDB demonstrated statistically significant improved overall survival with CCRT in stage II patients diagnosed with NPC in the United States. This is the only study to show benefit from the addition of chemotherapy to radiation in the US population. Even though older patients with high comorbidity scores and positive lymph node involvement had worse prognosis, our analysis suggests that even these groups did benefit from the addition of chemotherapy to radiation. A prospective randomized study would be the ideal solution to identify appropriate stage II NPC patients for CCRT, but such a study may not be feasible in the US population. In the absence of such data, results from our retrospective study could provide guidance to the practicing clinician when discussing the benefit of adding concurrent chemotherapy to radiation for patients with stage II NPC.

## CONFLICT OF INTEREST

Janakiraman Subramanian, M.D., M.P.H, Research Support—Biocept and Paradigm; Advisory Board—Alexion, Astra‐Zeneca, BMS, Boehringer‐Ingelheim Pfizer; Speaker Bureau—Astra‐Zeneca, Boehringer‐Ingelheim, Lilly; All other authors have no potential conflicts of interest to disclose
